# Antibiotic prophylaxis in dental implant surgery - A qualitative study on the attitudes and routines of Swedish dentists

**DOI:** 10.1186/s12903-025-07064-1

**Published:** 2025-11-20

**Authors:** Palwasha Momand, Bengt Götrick, Kristofer Hansson

**Affiliations:** 1 Department of Oral Diagnostics, Faculty of Odontology, Malmö University, Malmö, SE-20506 Sweden; 2 Department of Orofacial Medicine, Faculty of Odontology, Malmö University, Malmö, Sweden; 3 Department of Social Work, Faculty of Health and Society, Malmö University, Malmö, Sweden

**Keywords:** Antibiotic prophylaxis, Dental implant surgery, Antibiotic resistance, Qualitative study

## Abstract

**Background:**

Antibiotic resistance is a critical global health challenge, necessitating judicious antibiotic use across all medical disciplines, including dentistry.

**Methods:**

This qualitative study explored the attitudes and decision-making processes of Swedish dentists regarding antibiotic prophylaxis in dental implant surgery. A qualitative inductive research design was used to conduct semi-structured interviews with 17 dentists across Sweden. Purposive and snowball sampling ensured diverse perspectives.

**Results:**

Three key themes emerged: Perception of antibiotic resistance, Attitudes on antibiotic prophylaxis, and Need for clear guidelines. Participants demonstrated an awareness of antibiotic resistance as a significant public health concern but often perceived it as abstract compared to the immediate risks of postoperative infection. Their antibiotic prophylaxis practices varied widely, influenced by patient-specific factors, procedural complexity, and personal clinical experience. While some advocated selective use of antibiotic prophylaxis for high-risk cases, others routinely prescribe it to minimize complications, reflecting an ongoing tension between patient safety and antibiotic stewardship. A common frustration among dentists was the lack of evidence-based guidelines, which left decision-making subject to individual interpretation and contributed to practice variability. This underscores the need for standardized protocols that balance effective infection prevention with minimal antibiotic use.

**Conclusions:**

This study shows that while dentists recognize the importance of addressing antibiotic resistance, this awareness often feels secondary to the immediate clinical risks of postoperative infections. Practice around antibiotic prophylaxis varies depending on individual clinical judgment, surgical protocol, patient factors, and the absence of clear, evidence-based guidelines. The study highlights the need for evidence-based guidelines that balance patient safety and antibiotic stewardship.

**Supplementary Information:**

The online version contains supplementary material available at 10.1186/s12903-025-07064-1.

## Background

Antibiotic resistance is a critical global health challenge, contributing to substantial morbidity and mortality worldwide [1]. According to the World Health Organization (WHO), antibiotic resistance was directly responsible for approximately 1.27 million deaths globally in 2019, with an additional 4.95 million deaths linked to drug-resistant infections [2]. The misuse and overuse of antibiotics are primary drivers of this phenomenon, leading to the development of resistant bacteria [3]. This issue transcends economic welfare, threatening medical advancements across the globe [4].

Beyond its human toll, antibiotic resistance also imposes significant economic burdens, with additional healthcare costs projected to reach up to USD 1 trillion by 2025 [3]. Addressing this challenge requires a multifaceted approach, including accurate diagnostics, effective treatment of infections, correct use of antibiotic prophylaxis, the promotion of research and development for new medicines, and a deeper understanding of how healthcare workers use and perceive antibiotics [4]. The societal implications of antibiotic resistance have emerged as a significant public health crisis, prompting dystopian narratives about its consequences. An analysis of reports from various organizations highlights the role of imagination in framing the narrative around this crisis, emphasizing the need for a collective understanding of antibiotic resistance as a societal threat, rather than just an individual health issue [5].

In dentistry, antibiotics are used for several indications, both to treat ongoing infections and to prevent infections that can occur in conjunction with a specific treatment [6]. A common perception is that dentists in Sweden, as well as in other countries, prescribe more antibiotics than necessary for treating infections and for prophylaxis [7, 8]. Dental implant placement is a common surgical procedure for replacing missing teeth. Despite the generally high success rate of dental implants (90%–95% survive without issue for 15 years or longer), failures do occur [9]. One potential cause of early implant failure is bacterial contamination during implant placement, leading to an infection that necessitates removal of the implant [10, 11]. To mitigate this risk, various prophylactic methods, including antibiotics, have been used. However, the use of antibiotics for preventing dental implant failures remains a topic of debate. There are conflicting recommendations in consensus reports and systematic reviews. Most recommend routine antibiotic prophylaxis for implant surgery [12–16], while some advocate that antibiotic prophylaxis provides such a small risk reduction that it should be avoided in simple cases in healthy patients [17–20].

Every course of antibiotics contributes to the selection of resistant bacterial strains, allowing them to outcompete antibiotic-sensitive bacteria [21]. Given that antibiotic prescriptions in dental care contribute to the development of antimicrobial resistance, dentists *must* exercise caution and be as restrictive as possible in their use of antibiotics [22]. To reduce an overly liberal use of antibiotic prophylaxis, an understanding of how dentists reason and relate to antibiotic use and antibiotic resistance is necessary. By gaining insight into how dentists think about and justify their use of antibiotic prophylaxis, we can identify key areas for intervention and education, ultimately shaping more responsible prescribing behaviours. This aligns with the One Health approach, which recognizes that antibiotic resistance is not only a clinical issue but also a complex challenge spanning human, animal, and environmental health [3]. In their recent questionnaire-based study, Becker et al. [23] found a concerning trend of a liberal antibiotic use in connection with dental implant surgery, despite respondents being well aware of the antibiotic resistance issue. Well-performed qualitative studies that can lead to more insightful understanding of the topic are few, and more are needed.

## Methods

### Aim

The aim of the present study is, in our current context of growing risk of antibiotic resistance, to broaden our understanding of dentists’ attitudes on use of antibiotic prophylaxis in conjunction with dental implant surgery, and to explore how dentists’ reason about its use, including the contextual factor that influence their decision-making.

### Study design

This study employed a qualitative research design based on an interpretivist paradigm, aiming to explore the attitudes, reasoning, and contextual influences of dentists’ decisions regarding antibiotic prophylaxis. An inductive approach to qualitative content analysis, as described by Graneheim and Lundman [24], with data gathered through one-on-one, semi-structured interviews. This design was chosen to allow for a nuanced understanding of dentists’ subjective experiences and perspectives, which would not be possible through qualitative methods. For reporting guidelines, we chose to use The Consolidated Criteria for Reporting Qualitative Research (COREQ) [25].

### Settings and participants

The inclusion criteria were general practicing and specialized dentists who regularly perform dental implant surgery, defined as conducting at least 50 implant surgeries per year. Purposive sampling was used to select participants capable of offering insightful descriptions aligned with the study’s objectives. To approach an even distribution of participants across the occupational groups and a proportional mix of males and females, we also used snowball sampling [26]. Participants were initially identified through professional networks, dental associations, and university-affiliated clinics. Potential participants were approached personally, via email or telephone, informed about the study’s purpose, and invited to participate in a semi-structured interview. Those who expressed interest received written information and provided informed consent prior to participation. Informed consent to participate was obtained from all participants before data collection commenced. In the snowball sampling phase, enrolled participants were encouraged to suggest colleagues who met the inclusion criteria and might be interested in contributing to the study. The participants compromised 17 dentists from three occupational groups: oral and maxillofacial surgeons (2 males and 4 females), periodontists (2 males and 2 females) and general dentists who routinely preform dental implant surgery (4 males and 3 females). Clinical work experience ranged from 7 to 43 years (median 21). Participants worked in various cities around Sweden: from larger cities to smaller ones, from north to south. They were employed in the Swedish Public Dental Service, hospitals, and universities, or in private dental care. Our purposive sampling also involved factors such as years of experience, practice settings (e.g., public dental service, private dental care), specialization, and location to ensure a diverse range of perspectives. There were 18 dentists approached, whereof 17 participated and one could not take part due to time constraints. No participants withdrew after interviews had been scheduled or conducted.

### Interview guide

An interview guide was specifically developed for this study with input from all authors. The guide was designed to explore dentists’ attitudes, beliefs, and practices related to the use of antibiotic prophylaxis in dental implant surgery. The first author (a dentist) conducted in-depth, one-on-one interviews with the participants to explore their experiences and perspectives regarding the use of antibiotic prophylaxis in dental implant surgery. Additionally, the interviews examined their attitudes toward the risks associated with using antibiotics versus not using them. The interview guide comprised the following questions: *“Can you describe the last time you decided to administer antibiotic prophylaxis during implant surgery?”*,* “Do you believe that antibiotic prophylaxis is necessary in implant surgery?”*,* “Do you follow any guidelines when administrating antibiotic prophylaxis in conjunction with implant surgery”*,* “What risks do you believe are associated with the use of antibiotics?”*,* “How do you address this risk in your daily practice?”*,* “Do you discuss antibiotic use and the risk of antibiotic resistance with your colleagues?”*,* and “How does your knowledge about antibiotic resistance influence your decisions to use antibiotics?”*. When needed, the questions included prompts, such as Can you explain more about what you mean? Why do you think that is? What did you mean by …? The interview guide was pilot tested before the beginning of the study. We decided to include the pilot data in the study because, other than adding more prompt questions to clarify what the participants were saying, the interview guide required no substantial changes.

The interview guide is available as a supplementary file to this article.

### Data collection

The interviews took place over a 4-month period, from March to June 2024. We used Zoom and Teams for video meetings and occasionally visited nearby clinics for in-person meetings. Recruitment and interviews were conducted consecutively in order to achieve data saturation and, also, an even distribution between the three occupational groups. All interviews were audio recorded with the participants’ consent and transcribed verbatim to ensure accurate capture of all responses. The first author transcribed four of the interviews; the rest were sent to a transcription company. The interviews lasted between 13 and 46 min (median 33). A professional, bilingual translator in the dental field translated the quotations from Swedish to English. The authors considered data to be saturated when signs of redundancy began to emerge [27]. After the twelfth interview, interview responses became repetitive, so we decided to interview five more participants to ensure full saturation and an even distribution of participants. The interview transcripts were not returned to participants for review or correction, as member checking was not part of the study design. The interviewer (female dentists and researcher) knew of some participants through professional networks but did not have personal relationships with them. Participants were informed of her role as a dentist and researcher, and of her motivation to investigate antibiotic use in implant surgery. These factors may have influenced participants’ responses, and reflexivity was maintained through journaling and team discussions within the research group.

#### Data analysis

The first step was data immersion, where all authors read all transcripts multiple times. This familiarization process allowed the researchers to gain a holistic understanding of the content before beginning formal coding. In this stage, we made preliminary notes to capture initial thoughts and observations. The first author conducted the initial analysis, supported by the collaborative input of the second author (an experienced dentist and researcher) and the third author (an ethnographic researcher specializing in culture analysis). Transcribed data were organized and processed in NViVo 15. We used the inductive approach to qualitative content analysis described by Graneheim and Lundman [24]. The next step was identifying and extracting meaning units. Meaning units were defined as sentences or paragraphs related to each other through content and context. These meaning units were then condensed without losing their content and labelled with codes. This process was iterative and involved going through the transcripts line by line to ensure that no significant data had been overlooked.

When the coding process was completed, similar codes were grouped into categories. These categories represented broader concepts or themes that emerged from the data. Relationships between categories were explored, and sub-categories were identified to provide a more nuanced understanding of the data [28, 29]. We then identified themes by synthesizing the categories into broader patterns that encapsulated the core meanings and insights drawn from the data. Categories represent descriptive groupings of coded content, while themes are higher-level abstractions capturing underlying meaning across categories. Themes are more abstract than categories and provide a deeper understanding of the dentists’ attitudes and how their attitudes resonate with their current knowledge of antibiotic prophylaxis and its risks.

The initial themes were reviewed and refined in team discussions. This step ensured that the themes represented the data as accurately as possible and were not merely reflective of preconceptions [28]. Discrepancies or ambiguities in the themes were resolved through consensus or by returning to the data for clarification. Reflexivity was maintained throughout the study, with researchers continually reflecting on how their own background, biases, and assumptions might influence the analysis. Reflexive journaling and regular discussions among the research team helped mitigate potential biases and enhanced the transparency of the research process.

**Fig. 1:**
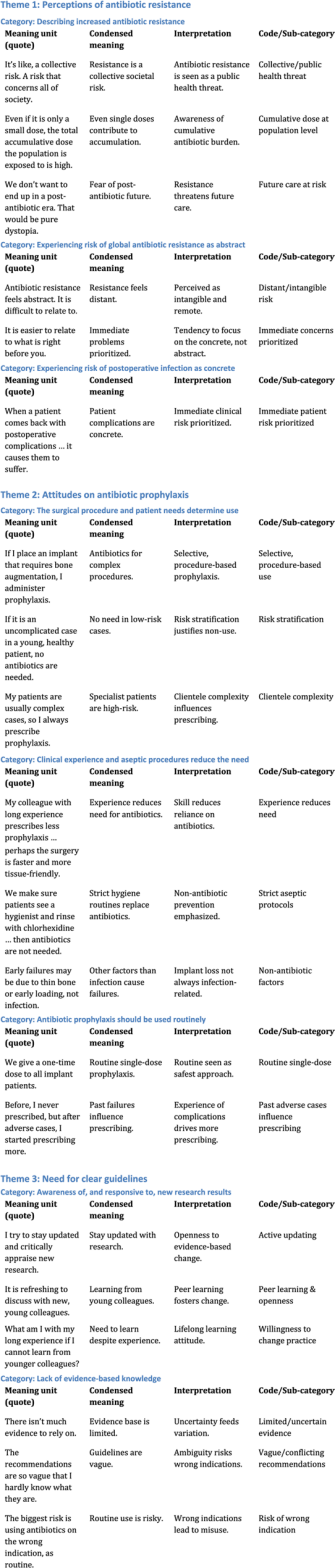
Detailed Coding Tree

As a final step, the identified themes were described in narrative form, with direct quotes from the interviews used to illustrate each theme [29]. This approach provided a rich, contextualized understanding of the dentists’ attitudes towards antibiotic prophylaxis in dental implant surgery. Each theme was discussed in detail, highlighting its relevance, the underlying factors, and how it connects to the broader context of dental practice, the lack of clear guidelines to adhere to, and antibiotic stewardship. The coding tree developed during analysis is provided as a supplementary material (Fig. 1).

## Results

Three themes were constructed through an iterative interpretive process from the interview data during analysis. The themes capture the essence of the attitudes and approaches of the dentists regarding antibiotic prophylaxis use in conjunction with dental implant surgery: Perceptions of antibiotic resistance, Attitudes on antibiotic prophylaxis, and Need for clear guidelines. Table 1 summarizes the themes and associated categories that emerged from the analyses. Direct quotations from the respondents are included to illustrate how the interpretation is grounded in the data.

**Table 1:** Overview of themes and categories

Theme	Category
*Perception of antibiotic resistance*	Describing increased antibiotic resistance
	Experiencing risk of global antibiotic resistance as abstract
	Experiencing risk of postoperative infection as concrete
*Attitudes on antibiotic prophylaxis*	The surgical procedure and patient needs determine antibiotic prophylaxis use
	Clinical experience and aseptic procedures reduce the need for antibiotic prophylaxis
	Antibiotic prophylaxis should be used routinely
*Need for clear guidelines*	Awareness of, and responsive to, new research results
	Lack of evidence-based knowledge

### Theme 1: perceptions of antibiotic resistance

Theme 1 captured how dentists perceive antibiotic resistance as a societal threat while navigating the ethical tension between this abstract risk and the immediate, concrete risks of postoperative infection for individual patients. This balancing act manifested in the following categories: *Describing increased antibiotic resistance*,* Experiencing the risk of global antibiotic resistance as abstract*, and *Experiencing the risk of postoperative infection as concrete.*

#### Theme 1, category 1: describing increased antibiotic resistance

The dentists spoke about the growing issue of antibiotic resistance in similar terms. They worried about contributing to the problem of antibiotic resistance through overprescribing or inappropriate use of antibiotics, which could exacerbate resistance patterns and impact overall public health. One dentist expressed:

It’s like, a collective risk. A risk that concerns all of society, not only the person whose bacteria develop resistance.

Periodontist, male, clinical experience ≤ 15 years.

Many study participants also recognized that every prescription of antibiotics contributes to the broader issue of antibiotic resistance. Some viewed this in a broader time perspective:

I think a lot about antibiotic resistance, [that] even if it is only a small dose, the total accumulative dose that the population is exposed to is high.

Oral and maxillofacial surgeon, female, clinical experience ≤ 15 years.

Here, the perception was that, over time, the more antibiotics that are used, the greater the risk that bacteria will evolve resistance, making it increasingly difficult to treat infections effectively. This understanding underscores the importance of using antibiotics judiciously and only when absolutely necessary to help mitigate the risk of resistance and preserve the effectiveness of antibiotics. One dentist expressed it this way:

Antibiotic resistance at the population level is a very important factor. It is clear that a single dose can contribute to the development of resistance.

General practising dentist, male, clinical experience ≤ 15 years.

Several participants noted that while a single dose of prophylactic antibiotics may seem insignificant, the cumulative effect of antibiotic prescriptions over time can lead to a substantial increase in overall antibiotic use on a population scale. This observation highlights a critical concern regarding the long-term implications of routine prophylactic use.

The participants expressed a need for greater awareness of how even individual prescriptions can collectively impact public health. More frightening images of the future also emerged:

The greatest risk of all that I first think about is, of course, the development of resistant bacteria. We don’t want to find ourselves in a post-antibiotic era. That would be pure dystopia. And we are rapidly heading there, aren’t we?

General practising dentist, female, clinical experience > 15 years.

The concern here focuses on the future and how society is moving toward a more dystopian state - a post-antibiotic era - where the effectiveness of antibiotics has diminished. This type of narrative is also found in other social contexts [5].

#### Theme 1, category 2: experiencing the risk of global antibiotic resistance as abstract

Several participants stated that the perception of growing global antibiotic resistance often feels abstract and distant, as it involves complex, long-term trends that aren’t always visible in daily practice. For many, the issue seemed remote because its impact unfolds gradually and on a global scale, rather than through direct, tangible experiences. The data revealed that dentists often experience a certain distance to this issue of antibiotic resistance because, unlike visible dental problems, it’s not something they encounter directly in their day-to-day practice.

The abstract nature of antibiotic resistance—its slow, systemic development and global implications—seems to make it feel less immediate or relevant to their specific role in patient care. While they understood it as a real public health threat, its effects were rarely visible in the short-term outcomes they saw with patients, leading to a sense of detachment. For many, the antibiotic resistance crisis felt remote from the concrete, hands-on postoperative complications they deal with, like infections or implant losses, making it easy to overlook the broader impact of prescribing antibiotics. One dentist reflected how things close in time were easier to relate to than things that were far away:

Still, it is difficult to always be considering a risk like that, which lies in a distant future and is difficult to imagine. It is just difficult to relate to. It is easier to relate to what is right before you.

General practising dentist, female, clinical experience > 15 years.

Viewing antibiotic resistance as a form of abstraction is also something that the dentists themselves reflectively related to in the interviews. As one dentist said:

Antibiotic resistance is a problem that, somehow, is very distant. It feels abstract. It is difficult to think about such a risk as something important and dangerous.

Oral and maxillofacial surgeon, female, clinical experience ≤ 15 years.

#### Theme 1: category 3: experiencing the risk of postoperative infection as concrete

In the interviews it became clear that, for dentists, it’s often more straightforward to focus on treating a patient’s immediate issues—like alleviating pain or preventing infection—than to weigh the broader, long-term risks of antibiotic resistance. When faced with a patient in discomfort or an infection that could complicate healing, prescribing antibiotics was highlighted as the quickest solution. The data revealed that this emphasis on direct care made it challenging for dentists to consistently prioritize antibiotic resistance risks in their day-to-day decision-making. One dentist reasoned that it is much easier to acknowledge the patients sitting in front of you and consider the risk of them returning with postoperative complications than to recognize the risk of antibiotic resistance:

It is difficult to consider the abstract and the concrete at the same time. What lays a long way in a distant future and what is sitting before you and suffering, and needs your help and time. When a patient comes back with postoperative complications, [the concrete] causes them to suffer.

Periodontist, male, clinical experience > 15 years.

This reflection illustrated how the abstract nature of antibiotic resistance creates a phycological distance that complicates clinical decision-making. While dentists intellectually understand the gravity of antibiotic resistance, they perceive limited agency in addressing this collective threat compared to their immediate ability to prevent concrete postoperative complications in individual patients. Consequently, the abstract nature of this risk can sometimes dilute the perceived immediacy of addressing antibiotic resistance.

### Theme 2: attitudes on antibiotic prophylaxis

A central theme in the interviews was the decision-making process concerning use of antibiotic prophylaxis in conjunction with dental implant surgery. This was an issue about which all interviewees had much to say. In this interview narratives, three categories emerged: *The surgical procedure and patient needs determine antibiotic prophylaxis use*, *Clinical experience and aseptic procedures reduce the need for antibiotic prophylaxis*, and *Antibiotic prophylaxis should be used routinely.*

### Theme 2, category 1: the surgical procedure and patient needs determine antibiotic prophylaxis

The interview data revealed an understanding and awareness in some participants that using antibiotic prophylaxis selectively was important and should be based on an evaluation of each individual’s situation, rather than making routine use standard for all patients. Many considered that selective, procedure-based use of antibiotic prophylaxis was highly important. The common ground for many of the dentists was that they considered antibiotic prophylaxis to be necessary in procedures that involved bone augmentation or in sinus lift surgery.

In general, you could say that if I am placing an implant that requires use of bone augmentation material, then I administrate antibiotic prophylaxis. The patient might even have diabetes or [a condition that lowers the patient’s immune response]. But if it is a common implant operation, like just placing a screw, then I don’t give [the patient] an antibiotic prophylaxis.

General practicing dentist, male, clinical experience ≤ 15 years.

Also, other dentist had similar reasoning:

It is those patients who have had bone transplantation, for example, and those patients with factors that mean they have worse preconditions for healing, a somewhat delayed ability to heal.

Oral and maxillofacial surgeon, female, clinical experience > 15 years.

One specialist indicated that he routinely administrates antibiotic prophylaxis to all of his patients, primarily because their implant surgery is usually complex. He noted that if he were treating patients undergoing low-risk procedures, his approach to prophylaxis would likely differ:

I always prescribe antibiotic prophylaxis for all types of implant operations to my patients. My patients are patients who have been referred to a specialist clinic for a reason. If it had been an uncomplicated operation without bone augmentation and such, on a young and healthy individual, I may have reasoned differently. But I don’t have such patients.

Periodontist, male, clinical experience > 15 years.

This reasoning may suggest that the type of patient clientele a dentist treats—considering both their individual health profiles and the complexity of the surgeries—influences the dentist’s perception of antibiotic prophylaxis use, particularly in balancing the need to prevent postoperative infections with the potential risks associated with antibiotic resistance. It highlights how clinical decisions may be shaped by a combination of patient-specific factors, procedural risks, and broader considerations of antibiotic stewardship.

#### Theme 2, category 2: clinical experience and aseptic procedures reduce the need for antibiotic prophylaxis

Many of the dentists, especially those with long clinical experience, explained that, when making clinical decisions, their own experience was something they trusted and relied on. The dentists claimed that such reliance on personal expertise is largely developed through years of hands-on practice and direct patient interactions, enabling them to observe firsthand the outcomes of either administering or refraining from antibiotic prophylaxis in conjunction with dental implant surgery. Such experiential knowledge is often invaluable, particularly in situations where empirical evidence is lacking or inconclusive.

The interview findings suggest that more experienced dentists tend to perform dental implant surgery with greater precision and efficiency, which can reduce the need for antibiotic prophylaxis. Our data revealed that many dentists believe that skilled clinicians are better equipped to handle complex surgical procedures, minimize tissue trauma, and manage potential complications during surgery. The long refinement of technique leads to lower rates of early implant failure, reducing reliance on antibiotics for infection prevention. Hence, experienced dentists may administer antibiotics more infrequently than dentists with less experience. Their practices were thus more closely aligned with current perceptions of antibiotic stewardship to combat the rise of antibiotic resistance. One oral and maxillofacial surgeon described a colleague who had long experience:

One of my colleagues at the clinic has worked many, many years with implants, he does not prescribe as much antibiotic prophylaxis as I do. And he … of course he also has failures, but I imagine that it is something about experience. Perhaps the operation takes less time, or he can operate in a, well, a more tissue-friendly way or something, which perhaps makes him not need to prescribe antibiotic prophylaxis as often. And I can also imagine that experience plays a role for surgeons, and also duration of the operation, most likely.

Oral and maxillofacial surgeon, male, clinical experience ≤ 15 years.

The dentists believed that by optimizing the septic environment—through measures such as strict sterilization of instruments, effective hand hygiene, and maintenance of a clean surgical field—they could create conditions that minimize the introduction of pathogens during the procedure. Such meticulous attention to aseptic technique could lead to a reduced reliance on antibiotic prophylaxis, as the likelihood of post-operative infections diminishes. Thus, in the long term, adopting robust septic protocols not only improves patient safety but also reduces the need for antibiotics. As one dentist explained:

We have a protocol where we always make sure that the patients have seen a dental hygienist and [that they] also rinse with chlorhexidine [for] five days before the operation and five days following the operation, with these patients, I don’t think that there is a need [to prescribe antibiotics] since we maintain such good hygiene routines, also during the operation.

General practicing dentist, male, clinical experience ≤ 15 years.

Here, the interviewee describes how ensuring that patients receive necessary preventative treatment to optimize their oral health can lead to more favourable outcomes.

#### Theme 2, category 3: antibiotic prophylaxis should be used routinely

Dentists who consistently administer antibiotic prophylaxis for dental implant surgeries often reasoned that it served as a precautionary measure to prevent potential infections, which could be costly and difficult to manage if they occur. Many felt that, given the complexity of implant procedures and the possibility of complications, prophylaxis minimizes risks for the patient and improves surgical outcomes. They may also have believed that a single course of antibiotics was unlikely to contribute significantly to resistance issues, particularly when weighed against the immediate need to protect the surgical site. For some, it was a matter of ensuring patient safety and satisfaction, especially if they felt that avoiding infections takes priority over long-term resistance concerns. One dentist expressed how he has gone from prescribing a week-long prophylactic regime to now giving one preoperative dose to all of his patients:

We do only one-time doses [of antibiotics] with all implant patients. I do, in any case. At the beginning of time, when I began with this, last century, they were given a week-long course afterwards.

Oral and maxillofacial surgeon, male, clinical experience > 15 years.

One of the dentists reflected on her journey of the opposite sort, from not administrating antibiotic prophylaxis during dental implant surgery to re-evaluating her approach based on personal experience. Initially hesitant, she has since observed outcomes that prompted her to adopt a more lenient perspective regarding prophylactic administration. This shift in her mind-set illustrated a growing recognition of the nuanced role antibiotics can play in patient care, highlighting the importance of adapting clinical practices based on evolving evidence and first-hand experiences:

I’ll put it like this: before, I never prescribed it, but now, I have begun to give a little more. And that is because I have had a few cases where I … have had … when I removed the sutures … observed somewhat abnormal healing where I haven’t really … where it has been very obvious … straightforward cases …. And then I have spoken with other colleagues, to see what they thought. So I spoke with one colleague who doesn’t work here, but a periodontist who works privately. And he says that, yes, he always prescribes antibiotic prophylaxis [during implant surgeries].

Periodontist, female, clinical experience > 15 years.

### Theme 3: need for clear guidelines

The dentists in this study all agreed that the effectiveness of antibiotic prophylaxis in conjunction with dental implant surgery is unclear, mainly due to a lack of well-conducted clinical studies and varying interpretations of available research results. Factors such as the patient’s overall health, the specific surgical techniques used, and experience of the operator can all influence the outcome. Many of the participants spoke about how clear guidelines are needed and emphasized that there is lack of solid evidence-based consensus on this issue.

The third theme comprises the following categories: *Awareness of*,* and responsive to*,* new research results* and *Lack of evidence-based knowledge*.

#### Theme 3, category 1: awareness of, and responsive to, new research results

The material revealed that many dentists emphasized a responsiveness to new evidence as a crucial factor in their daily practices because it ensures that they stay current with the latest advancements and best practices. Incorporating new research into clinical practice allows dentists to provide the most effective, evidence-based care, improving patient outcomes and safety. Many dentists agreed that staying updated with emerging evidence helps retire outdated practices, enhances treatment efficacy, and fosters continuous improvement in dental care standards. As one dentist said:

Sometimes, many wise heads need to be put together in order to get a better understanding. It is refreshing to discuss with new, young colleagues. Those who perhaps have just graduated or just begun as an intern. Such a “breath of fresh air”, they gladly share the latest that they have learned in their education.

Periodontist, male, clinical experience > 15 years.

The data indicated that more experienced specialists often show a strong interest in learning from their younger colleagues. This willingness to engage with emerging perspectives highlights a collaborative spirit within the profession, suggesting that seasoned practitioners value fresh insights and innovative approaches brought by newer generations. Such interaction can foster continuous professional development and enhance overall patient care in dental practice. As one experienced periodontist expressed:

You can have long experience and learned much by having worked clinically for a long time. But what then? What am I with my long experience if I cannot learn anything new from those younger than I?

Oral and maxillofacial surgeon, male, clinical experience > 15 years.

#### Theme 3, category 2: lack of evidence-based knowledge

Several participants felt the lack of evidence-based knowledge was a problem since this lack makes it challenging for clinicians to establish standardized protocols and leaves room for debate on the necessity and efficacy of antibiotics in preventing infections and early implant failures. This leads to dentists having to rely on their clinical judgement and individual patient assessments. One dentist claimed that the current existing recommendations were very vague:

There are recommendations, but they are so vague that … I hardly know what the recommendations are, if I should be honest.

General practicing dentist, male, clinical experience ≤ 15 years.

The below also reveals the ambiguity that the interviewees often face due to the lack of clear, standardized guidelines:

There is *way* too much wiggle room for personal interpretation of the recommendations we have today. We lack clear guidelines that we, as clinicians, should be able to follow without doubts. Because of this, there is space for each clinician to do, more or less, as one considers best, whether it is based on your instructors or, perhaps, that you follow the latest research results.

General practicing dentist, female, clinical experience > 15 years.

## Discussion

These study findings shed light on the intricate and multifaceted decision-making processes of dentists regarding the use of antibiotic prophylaxis in dental implant surgery. The themes that emerged—Perceptions of antibiotic resistance, Attitudes on antibiotic prophylaxis, and Need for clear guidelines—collectively highlight the complexity of navigating the ethical, clinical, and public health dimensions of antibiotic use in modern dental practice. These themes not only reveal the challenges that dental professionals face today, but they also underscore the broader issues related to antibiotic stewardship in healthcare.

### Awareness of and attitude on antibiotic resistance: ethical tension in clinical practice

The dentists’ awareness of antibiotic resistance reflects one of the many layers of complexity—balancing the immediate needs of individual patients with the long-term consequences of antibiotic overuse for society. The dentists in this study demonstrated a sophisticated understanding of the risks posed by antibiotic resistance, expressing concerns about contributing to the global crisis through unnecessary prescriptions. The WHO has repeatedly emphasized the need for healthcare professionals to act as stewards in the effort to abolish antibiotic resistance [30], and the dental profession must also be involved in this effort. However, a common experience among the participants was that they found the risk of contributing to antibiotic resistance to be abstract and distant, particularly when compared to the concrete risks of postoperative infections or implant loss. This finding reflects the broader challenges in public health, where long-term and widespread threats, such as antibiotic resistance, can seem remote or less pressing compared to the tangible clinical risks that present themselves in day-to-day practice. As Ouellet et al.(2021) noted, healthcare professionals face a dilemma: while they are aware of the long-term societal impacts of antibiotic overuse, they often feel compelled to prioritize the prevention of direct complications in their patients [31].

Antibiotic resistance is a significant societal challenge, profoundly impacting public health and healthcare systems globally. Recent research exploring public perceptions in Sweden, revealed that individuals often lack awareness regarding the implications of their antibiotic use, highlighting a critical need for education on responsible consumption and its broader health consequences​ [32]. They concluded that societal factors, including cultural beliefs and practices, play a vital role in shaping antibiotic prescribing behaviours. We believe this also applies to those professions - dentists and doctors - who handle antibiotics in their daily work. Educational initiatives aimed at altering public perceptions about antibiotic efficacy and the dangers of resistance are essential to mitigate these risks [32].

Unlike many other diseases that are viewed more individually within the patient’s body, resistant bacteria are considered a threat to society and the collective. The statement regarding antibiotic resistance as a societal threat rather than an individual health issue is well-supported in the literature [5, 33–36]. Antibiotic resistance is increasingly viewed as a collective problem, emphasizing that the misuse of antibiotics affects not just the individuals who overuse them but society at large. This perspective is crucial as it encourages a shared responsibility among healthcare providers, patients, and policymakers to combat resistance [37]. For instance, the Yee et al. report (2020) discusses how public health education and policy interventions are necessary to address antibiotic misuse, highlighting that everyone must contribute to responsible antibiotic use to protect public health [38].

The current study revealed that some participants were of the view that antibiotics can have a significant impact on patients at the individual level by potentially leading to bacteria becoming resistant to commonly prescribed antibiotics. In this scenario, patients would experience more severe or recurrent infections that are harder to manage and require more aggressive or alternative treatments. Managing resistant infections often results in higher healthcare costs and greater strains on both patients and the healthcare system itself [2, 39]. In this way, it is a narrative that not only connects the individual prescription with a global threat, but also links the global threat to a future patient who will not be helped by ineffective antibiotics.

### Approaches to antibiotic prophylaxis: balancing selectivity, clinical practice, and routine use

The focus on selective, individualized use of antibiotic prophylaxis reflects an ambition to balance patient-centred care with wider public health concerns. Dentists in this study demonstrated a nuanced understanding of the need to tailor antibiotic prophylaxis based on the extent of the surgical procedures and the health status of the patients. However, this selectivity introduces a degree of variability in clinical practice, with practitioner interpreting risk factors differently.

In systematic reviews, such as those by Braun et al. (2019) and Jain et al. (2020), it is suggested that while selective antibiotic prophylaxis is beneficial for high-risk patients, its routine use in low-risk populations provides little added value [40, 41]. Yet, without clear and universally accepted guidelines, dentists are left to make decisions on their own and such decisions can vary significantly between different dentists. Studies show that antibiotics administered before surgery do help control bacterial load, which is desirable in surgeries requiring bone augmentation because these situations are more susceptible to infections that could jeopardize implant success [42].

Research claims that dental specialists tend to administer more antibiotic prophylaxis compared to general dentists. A study examining the prescribing habits of general dentists and oral and maxillofacial surgeons in Australia found that a higher percentage of specialists routinely prescribed antibiotics for procedures like dental implant placements, 72% of specialists compared to 62% of general dentists [43]. Specialists often handle more complex cases, such as patients with chronic diseases, extensive bone loss, or other complicating factors, which could explain this difference. The perceived risk of postoperative infections may be higher in these situations, prompting specialists to adopt a more liberal approach with antibiotic prophylaxis to mitigate potential complications. One dentist in our study explained that the reason he consistently administered antibiotic prophylaxis was because his patients typically required complex treatments. He added that if his patient base were different, he would likely approach the use of antibiotics differently.

The reliance on clinical experience, particularly among more senior dentists, adds another layer of complexity to the findings. Experienced dentists often use years of practice and patient outcomes to guide their decisions regarding antibiotic prophylaxis. This approach is understandable, especially in the absence of clear guidelines [44]. However, it can also introduce variability and perpetuate outdated practices.

As Becker et al. (2024) highlighted, clinical experience can sometimes lead to a departure from evidence-based practices, particularly if new evidence challenges established habits [23]. This theme underscores the need for continuous professional development and lifelong learning in dentistry to ensure that clinical experience is supplemented by the latest research and evolving guidelines.

### Uncertainty and need for clear guidelines: navigating ambiguity in practice

Perceptions among dentists are divided due to the weak evidence base surrounding antibiotic prophylaxis in dental implant surgery. No randomized, placebo-controlled clinical trials have yet found a significant difference in treatment outcome between antibiotic use and a placebo [45–52]. Several systematic reviews have reported that preoperative antibiotic prophylaxis significantly reduces early implant failure [14–16, 41, 53]. However, the reduction is small, resulting in a high NNT (number needed to treat) [53, 54]. Furthermore, a recently published systematic review concludes that the overall evidence regarding antibiotic prophylaxis remains inconclusive [55]. Such uncertainty underscores the complexity of antibiotic decision-making in dental implant procedures. Thus, this ambiguity has left many clinicians feeling uncertain about when antibiotic prophylaxis is truly warranted, leading to a wide range of practices, even within similar clinical environments [56, 57].

The uncertainty in clinical decision-making mirrors findings from other healthcare fields, where it could potentially lead to suboptimal patient outcomes [39]. The desire for clearer guidelines highlights how complex it is to integrate evolving research into clinical protocols, especially when research findings do not provide a definitive course of action. This complexity is exacerbated by the pressure on clinicians to prevent immediate patient complications, such as infections and implant loss, even when long-term health concerns, such as antibiotic resistance, should be taken into consideration.

### Complexity of the findings

Overall, the themes identified in this study reflect the inherent complexity of antibiotic prophylaxis in dental implant surgery. The decisions to prescribe antibiotics are not a simple clinical choice but are influenced by a range of factors, including type of surgery, patient risk profiles, and personal clinical experiences. Each of these factors interact in ways that complicate decision-making, leading to substantial variability in practice.

This complexity underscores a significant challenge within healthcare: the need to balance evidence-based medicine with personalized patient care, referring to the clinician’s approach to antibiotics in their interaction with individual patient. In the realm of dental implant surgery, practitioners face the delicate task of minimizing patient complications while also considering the long-term implications of antibiotic use for antibiotic resistance. This dual focus necessitates careful decision-making, as dentists must weigh the immediate benefits of prophylactic antibiotics against the broader public health impact of antibiotic misuse [37, 38]. Such a balancing act is complicated by the lack of clear guidelines and the need to rely on personal judgment, which can vary widely between dentists. Addressing this complexity requires not only clearer guidelines but also a more nuanced understanding of how different factors—clinical, ethical, and experiential—interact in the decision-making process [58].

### Limitations

Several limitations should be pointed out. First, sampling was confined to dentists practicing in Sweden, which may limit the generalizability of the findings to other countries with different healthcare systems, regulatory frameworks, and clinical practices. Additionally, while efforts were made to include a diverse range of participants in terms of experience and practice setting, the relatively small sample size (17 participants) may not have fully captured the spectrum of opinions within the profession. Although we continued interviewing until saturation was reached, the findings in the results specifically reflect the responses from the dentists who were interviewed. Another limitation is the potential for interview bias, as the interviewer was a dentist. The shared professional background could have influenced participant responses, potentially leading them to express views they believed would align with the interviewer’s perspective. Although steps were taken to minimize this bias, such as using open-ended questions and maintaining a neutral tone, future studies may benefit from using independent interviewers or anonymous methods to reduce any interview bias.

### Future research

The findings of this study suggest several areas for future research. Comparative studies across different regions and healthcare systems could help to elucidate how local practices and regulations influence antibiotic prophylaxis use in dental implant surgery. Further research should also focus on identifying which patient population benefits most from antibiotic prophylaxis and on evaluating the long-term impact of selective versus routine antibiotic prophylaxis strategies on both patient outcomes and antibiotic resistance. Longitudinal studies that track how changes in guidelines or stewardship programs affect prescribing behaviours would provide valuable insights into how education and policy affect clinical practice. Additionally, randomized placebo-controlled trials assessing the effect of antibiotic prophylaxis in different types of surgeries including bone augmentation, as well as comparing different prophylactic protocols, would be essential in developing more definitive, evidence-based guidelines for antibiotic prophylaxis in dental implant surgery. Furthermore, future work should integrate frameworks from Implementation Research (e.g. Theoretical Domains Framework [59, 60] and/or Consolidated Framework for Implementation Research [61]) to systematically identify barriers and facilitators influencing the adoption of evidence-based antibiotic prophylaxis guidelines. Such research could map contextual factors, identify cognitive behavioural barriers, develop and evaluate tailored implementation strategies. These approaches would address the core challenge identified in our study: translating guidelines into consistent clinical practice amidst competing priorities and ambiguity.

## Conclusion

This study aimed to understand dentists’ attitudes and decision-making regarding antibiotic prophylaxis in dental implant surgery amidst the growing concern of antibiotic resistance. The findings reveal that while dentists recognize the importance of addressing antibiotic resistance, this awareness often feels secondary to the immediate clinical risks of postoperative infections. Practices around antibiotic prophylaxis vary, influenced by individual clinical judgment, surgical protocol, patient factor, and the absence of clear, evidence-based guidelines. The study highlights the need for evidence-based guidelines that are based on a balance between patient safety and antibiotic stewardship.

## Supplementary Information

Supplementary Material 1. 

## Data Availability

All datasets used and/or analysed during the current study are available from the corresponding author on reasonable request.
