# Supplementary material for: Antibiotic prophylaxis in dental implant surgery - A qualitative study on the attitudes and routines of Swedish dentists
Source: BMC Oral Health. 2025 Nov 20;25:1807. doi: 10.1186/s12903-025-07064-1 (PMC12636202; doi:10.1186/s12903-025-07064-1)
Supplement: Supplementary file 1 — Supplementary Material 1. [file 12903_2025_7064_MOESM1_ESM.docx]

**Interview guide**

- Can you describe the last time you decided to administer antibiotic prophylaxis during implant surgery?
- Do you believe that antibiotic prophylaxis is necessary in conjunction with implant surgery?
- Do you follow any guidelines when administrating antibiotic prophylaxis in conjunction with implant surgery?
- What risks do you believe are associated with the use of antibiotics?
- How do you address this risk in your daily practice?
- Do you discuss antibiotic use and the risk of antibiotic resistance with your colleagues?
- How does your knowledge about antibiotic resistance influence your decisions to use antibiotics?
